# The Mobile Charging Vehicle Routing Problem with Time Windows and Recharging Services

**DOI:** 10.1155/2018/5075916

**Published:** 2018-10-08

**Authors:** Shaohua Cui, Hui Zhao, Hui Chen, Cuiping Zhang

**Affiliations:** ^1^School of Traffic and Transportation, Beijing Jiaotong University, Beijing 100044, China; ^2^MOE Key Laboratory for Urban Transportation Complex System Theory and Technology, School of Traffic and Transportation, Beijing Jiaotong University, Beijing 100044, China; ^3^Computing Center, Beijing Information Science & Technology University, Beijing 100192, China

## Abstract

For the environmental friendliness of the technology on battery electric vehicles, there is growing attention on it. However, the market share of battery electric vehicles remains low due to the range anxiety. As a remedy, the mobile charging services could offer charging service at any time or locations requested. For profitability of the services, the operator should route the charging vehicles in a more efficient manner. For this consideration, we formulate the mobile charging vehicle routing problem as a mixed integer linear program based on the classical vehicle routing problem with time windows. To demonstrate the model, test instances are designed and computational results are presented. In order to examine the change of the number of mobile charging vehicles and travel distance, sensitivity analyses, such as battery capacity and recharging rate, are performed. The results show that larger battery capacity, quicker charging rate, or higher service efficiency could decrease the number of mobile charging vehicles and total traveled distances, respectively.

## 1. Introduction

As an emerging technology for reducing petroleum consumptions and carbon dioxide emissions, the battery electric vehicles (BEVs) have been deployed increasingly in recent years due to the development of battery charging technology [1, 2]. As it was reported in 2013 that transportation accounts for over 60% of all petroleum consumptions in the United States, and 60% of the petroleum consumptions was imported [3], the energy conservation benefits of deploying BEVs could be expected. It was also evaluated that shifting from conventional gasoline vehicles to plug-in hybrid electric vehicles could reduce the gasoline consumptions by up to 52% of the petroleum import in the United States [4]. For the environment perspective, it was shown that transportation was responsible for 23% of the worldwide carbon dioxide emissions [5], and using BEV could even reduce carbon emissions by up to 60% in some cases comparing with conventional hybrid vehicles [6].

**Figure 1:**
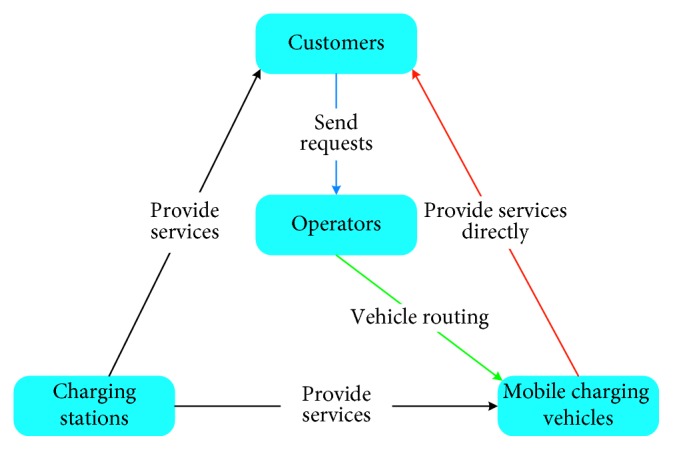
The mobile charging service flow chart.

Though the benefits could be confirmed for deploying BEVs, there is still a barrier for people to choose BEVs, which is range anxiety [7]. The range anxiety refers to the fact that BEV drivers fear for batteries running out of power en route due to the limited battery capacity of power [8, 9]. The inevitability of range anxiety prevents BEV drivers to take longer journey. As a remedy, the deployment of public charging infrastructure plays a critical role in the BEV market and promoting the adoption of BEV [10, 11]. But the supply of the public charging infrastructure is far from enough at present, especially in developing countries. Therefore, there are cases that BEV could breakdown because of the limited power en route. In the cases, rescue service should be provided for the breakdown BEVs. Note that for BEVs, it is not easy to refill the power as for the traditional gasoline vehicles. Nowadays, there is a special type of vehicle which is the mobile charging vehicle (MCV) that could provide charging services for BEV drivers with requests [12]. Furthermore, with the rapid development of mobile Internet, BEV drivers could submit their charging service requests via Apps on smartphones, such as Eyuechongdian [13]. As mentioned by Cui et al. [13], the operators arrange MCVs after the service requests are collected, such as: BEV identification, state of charge, location, charging time interval, etc. The detailed mobile service flow is shown in Figure 1. First, the customer sends a charging service request to the operator through the App. The operator will then arrange for MCVs to provide the service, but there will be two situations at this time where one situation is that if the car has enough power, it will provide the service directly to the customer (along the red line); otherwise, it will first go to the charging station to recharge its battery itself and then provide the service (along the black line). Therefore, the mobile charging service can be a good solution to the problem of insufficient battery power during car travel.

For the operators, if a number of charging service requests are received, problems may arise, including how many mobile charging vehicles are arranged to provide services, how to design the service path of each vehicle, etc. To address the problems mentioned above, this paper presents a mathematical model based on the vehicle routing problem with time windows (VRPTW).

In the literature, the VRPTW has been extensively studied by many researchers [14–18]. Concerning the widespread use of BEVs, based on the traditional VRPTW, Schneider et al. [19] studied the electric vehicle routing problem with time windows (E-VRPTW) where the limited operating range and the possibility of charging of BEVs at some stations were considered. But in the study of Schneider et al. [19], BEVs must be fully recharged at the stations they visited, which could cause damage to time windows and thus affect the service path. Therefore, Keskin and Çatay [20] proposed a new model—partial recharge strategy for electric vehicle routing problem with time windows (E-VRPTW-PR)—to find recharging strategies which could recharge BEVs partially and could charge the power required only to make the model more realistic.

As in our research, the mobile charging vehicles are also assumed to be electric powered, the model proposed in this paper is also built on the basis of E-VRPTW-PR [20]. But the goal of our research is to find the routing strategies for providing recharging services to BEV drivers with lack of power en route. Furthermore, in this study, we also incorporate the possibility of recharging at any available stations using an appropriate recharging scheme. Though it seems that from the operational research perspective, E-VRPTW is the vehicle routing problem where the vehicles consume electricity on the paths and could refuel at charging station, while actually, the problem studied here is on the mobile charging service, where the mobile charging vehicles can consume electricity on the paths and the service points. The aim of our paper is to address a distance effective routing strategies for the electric powered mobile charging vehicles, starting (or returning), from (or to) a common depot, in order to handle a set of charging requests by BEVs with lack of power en route at some specific time windows.

By formulating the electric charging vehicle routing problem as a 0–1 mixed integer linear program (MILP), we discuss the optimal routing and recharging strategy. As in reality, there could not be so many rescue requests at the same time, in this research, we solve the model using commercial solvers, and a set of small-sized instances is designed to demonstrate the proposed model.

Till now, two studies focused on the mobile charging service which was conducted by Huang et al. [12] and Cui et al. [13]. For the former, Huang et al. [12] used a queuing based analytical approach called “nearest job next,” while Cui et al. [13] used the location routing problem (LRP) to design the location of charging facility and the routing plan of MCVs simultaneously, which is different with our research. In fact, this paper aims to minimize the total distance of MCVs to complete all charging requests for BEV drivers in distributed locations, providing operators with the best path design. The contribution of this paper can be summarized as follows:

1. The mobile charging vehicle routing problem is formulated as an MILP based on the E-VRPTW-PR considering both limited operation ranges and possible recharging strategies
2. A set of benchmark instances is designed based on the work by Solomon [21]
3. Potential application of the model is discussed extensively

The remainder of this paper is organized as follows. In the following station, the major features of the mobile charging vehicle routing problem are illustrated through a simple example.  Section 3 formulates the model based on E-VRPTW-PR. Experimental results obtained on newly designed instances and sensitivity analyses are presented in Section 4.  Section 5 gives a short summary and potential applications of the proposed model.

## 2. Problem Descriptions

In the research, we concern a homogeneous fleet of mobile charging vehicles powered by rechargeable battery with fixed capacity that starts from/returns to a common depot to handle a set of charging service requests by the BEVs, which is lack of power and should be served inside specific time windows. Following Cui et al. [13], the aim is set as minimizing the total travel distance of the vehicles due to the neglect of traffic congestion. Unlike Keskin and Çatay [20], in this paper, the power consumption of MCVs is related to the distance traveled and the electricity demand of the customer nodes. And then, MCVs may choose to recharge their battery when the power is not enough where the battery is recharged at any quantity at charging stations. In this paper, we assume that the depot could also be used as a charging station and provide charging service for the mobile charging vehicles. For less recharging time en route, mobile charging vehicles return to the depot with an empty battery if it has been recharged en route, which means that the amount of charge of the MCVs is equal to the amount of charge they need.

**Figure 2:**
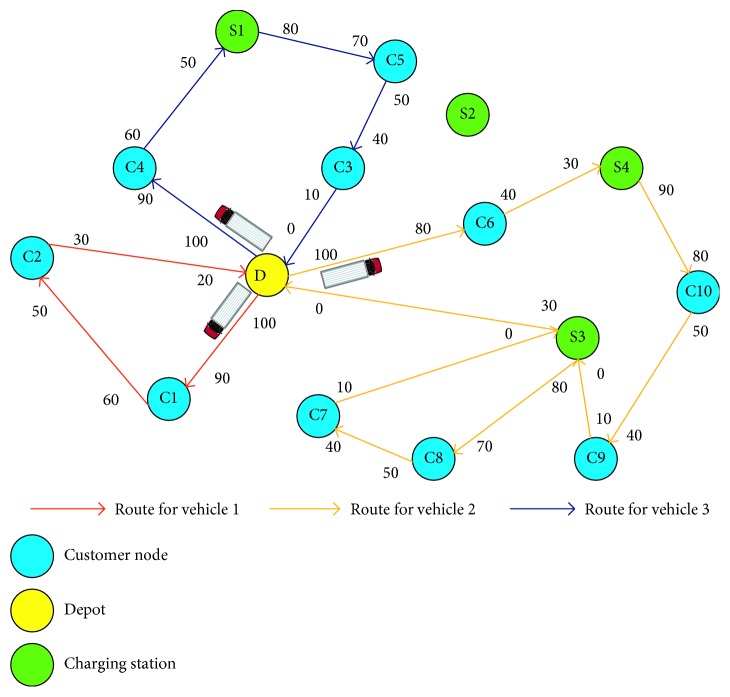
An illustrative example, the numbers on the branches is the state of charge of a BEV before it enters and leaves out the specified node.

For clearer description , we use a simple network adapted by Cui et al. [13] that is shown in Figure 2. This example involves ten customers (C1–C10), four charging stations (S1–S10), and one depot (D). From Figure 2, one can confirm that all services could be completed by 3 vehicles with 3 different service paths accordingly. The percentage values along the routes show the states of charge for each vehicle arriving at/departing from a customer node or a charging station. From the figure, we can find that before the vehicles 2 and 3 return to the depot, they visit charging stations en route and consume all power when they end their services, while vehicle 1 does not recharge the battery during service and returns to the depot with the remaining 20% of the battery power. Furthermore, for the charging stations, a charging station may be visited several times by the same (see S3) or different vehicles, and the stations may not necessarily be visited (see S2). For this example, the routes may not be optimal, which will be further calculated in next section by a MILP formulation.

## 3. The Model

To formulate the mobile charging vehicle routing problem, some basic assumptions could be listed below:

1. The terrain is flat, that is, no grades are considered, so the travel speed between every node is constant and given
2. The recharging rate is fixed and given as a constant *g*
3. The customer can accept the electricity provided by the mobile charging vehicles entirely, and the charging services are performed by a homogeneous fleet of mobile charging vehicles with fixed battery capacity *Q*

Let *V*={1,…, *N*} be a set of BEVs with requests, which can be seen as customer nodes in the network, and *F*={1,…, *M*} denote the set of recharging stations that may be visited more than once. Mathematically, the set of BEVs with requests *V* and the set of recharging station *F* can be seen as a subset of the node set. We denote *F*′ as the set of dummy nodes to permit several visits to each node in the set *F*. Let *V*′=*V* ∪ *F*′. For the depot, we set both 0 and *N*+1 to denote it, which are the same node. Note that every route starts at 0 and ends at *N*+1. For convenience, we set *F*_0_′=*F*′ ∪ {0}, *V*_0_′=*V*′ ∪ {0}, *V*_*N*+1_′=*V*′ ∪ {*N*+1}, and *V*_0,*N*+1_′=*V*′ ∪ {0} ∪ {*N*+1}. Let the set of links be *A*={(*i*, *j*) | *i*, *j* ∈ *V*_0,*N*+1_′, *i* ≠ *j*}, and then the problem could be defined on a complete directed graph *G*=(*V*_0,*N*+1_′, *A*). For each link, the distance *d*_*ij*_ and travel time *t*_*ij*_ are predetermined. For the mobile charging vehicle, the battery consumption rate is assumed to be constant *h*, and then every traveled link consumes *hd*_*ij*_ of the remaining battery. For each customer node, the positive service time *s*_*i*_, number of requests *u*_*i*_, and demand of charging *q*_*i*_ for each request, are given. It is obvious that the total amount of charging could be calculated as *u*_*i*_*q*_*i*_ at node *i* ∈ *V*_0,*N*+1_′. Furthermore, for each customer node, the time window is assumed to be predetermined also and it could be noted as [*e*_*i*_, *l*_*i*_]. As the mobile charging vehicle should recharge for its own battery at charging station *i* ∈ *F*′, so the recharging time for the mobile charging vehicle is associated with the recharging rate *g* and the state of charge *y*_*i*_ when the mobile charging vehicle arrives at node *i*. The state of charge for the mobile charging vehicle leaving node *i* is set to be *Y*_*i*_, which is obviously less than or equal to the battery capacity *Q*. In order to track the arrival time of the mobile charging vehicles, a decision variable *τ*_*i*_ is defined for each node *i*. Let *x*_*ij*_ be a binary decision variable for all *i* ∈ *V*_0_′, *j* ∈ *V*_*N*+1_′, *i* ≠ *j*, which takes value 1 if link (*i*, *j*) is traveled and 0 otherwise.

Then, the mobile charging vehicle routing problem could be formulated as a 0–1 MILP. For the readability of the paper, we list notations used in Table 1.

(1) $$\begin{array}{c} \min\underset{i\in V_{0}^{\prime },j\in V_{N+1}^{\prime },i\neq j}{\sum }d_{ij}x_{ij}, \end{array}$$

Subject to:

the flow constraints:

(2) $$\begin{array}{c} \underset{j\in V_{N+1}^{\prime },i\neq j}{\sum }x_{ij}=1,\;\forall i\in V, \end{array}$$

(3) $$\begin{array}{c} \underset{j\in V_{N+1}^{\prime },i\neq j}{\sum }x_{ij}\leq 1,\;\forall i\in F^{\prime }, \end{array}$$

(4) $$\begin{array}{c} \underset{i\in V_{N+1}^{\prime },i\neq j}{\sum }x_{ji}-\underset{i\in V_{0}^{\prime },i\neq j}{\sum }x_{ij}=0,\;\forall j\in V^{\prime }, \end{array}$$

the time constraints:

(5) $$\begin{array}{c} \tau_{i}+\left(t_{ij}+s_{i}\right)x_{ij}-l_{0}\left(1-x_{ij}\right)\leq \tau_{j},\forall i\in V_{0},\;\forall j\in V_{N+1}^{\prime },\;i\neq j, \end{array}$$

(6) $$\begin{array}{c} \tau_{i}+t_{ij}x_{ij}+g\left(Y_{i}-y_{i}\right)-\left(l_{0}+gQ\right)\left(1-x_{ij}\right)\leq \tau_{j},\;\forall i\in F^{\prime },\forall j\in V_{N+1}^{\prime },\;i\neq j, \end{array}$$

(7) $$\begin{array}{c} e_{i}\leq \tau_{i}\leq l_{i},\;\forall i\in V_{0,N+1}^{\prime }, \end{array}$$

the electricity constraints:

(8) $$\begin{array}{c} x_{ij}q_{j}u_{j}\leq y_{j}\leq y_{i}-{\text{hd}}_{ij}x_{ij}-q_{i}u_{i}x_{ij}+Q\left(1-x_{ij}\right),\forall i\in V,\;\forall j\in V_{N+1}^{\prime },\;i\neq j, \end{array}$$

(9) $$\begin{array}{c} x_{ij}q_{j}u_{j}\leq y_{j}\leq Y_{i}-{\text{hd}}_{ij}x_{ij}+Q\left(1-x_{ij}\right),\forall i\in F_{0}^{\prime },\;\forall j\in V_{N+1}^{\prime },\;i\neq j, \end{array}$$

(10) $$\begin{array}{c} y_{i}\leq Y_{i}\leq Q,\;\forall i\in F_{0}^{\prime }, \end{array}$$

(11) $$\begin{array}{c} 0\leq q_{i}u_{i}\leq Q,\;i\in V, \end{array}$$

binary variable:

(12) $$\begin{array}{c} x_{ij}\in \left\{0,1\right\},\forall i,\;j\in V_{0,N+1}^{\prime },\;i\neq j. \end{array}$$

The objective function minimizes the total travel distance in Equation (1). Equation (2) is the constraint for the flow at the customer nodes, which ensures that every customer could be visited only once by any mobile charging vehicle. Equation (3) is for the dummy nodes which guarantee the connectivity of visits to dummy recharging stations and restricts that each dummy node could be visited only once by any mobile charging vehicle. Constraint (4) is for flow conservation at intermediate nodes. Equation (5) is the time feasibility constraints for arcs leaving customer nodes and depot node with instance 0. Equation (6) does the same for arcs leaving recharging station. The constraint (7) enforces the time windows for every nodes. Equation (8) and Equation (9) keep track of state of charge of the battery for the customer nodes, charging dummy nodes, and deport, respectively, and ensure that the battery state of charge never falls below electric demand of next customer node. Equation (10) makes sure that the battery state of charge could not exceed its capacity. Equation (11) is the constraint for the demand of customer nodes, which sets the upper and lower bounds of the demand. Finally, Equation (12) defines the binary decision variables *x*_*ij*_.

**Proposition 1 .**

*In an optimal solution, if a mobile charging vehicle does not fill its battery itself when it leaves the depot, that isY*_0_^*∗*^ < *Q, then, when the same car leaves the depot full charged, that isY*_0_^*∗*^=*Q, the solution is also optimal*.

**Proof**

Because the recharging time at the depot does not delay the leaving time of the car (Equation (9)), therefore, when the car is fully charged, the solution is also optimal.

**Corollary 1 .**

*If there is one car not fully charged leaving the depot, that isY*_0_^*∗*^ < *Q, and the solution is optimal, then the problem has infinite multiple optimal solutions*.

**Proof**

By contradiction, we assume, in the optimal solution, the charge of one vehicle is ${\bar{Y}}_{0}$, and is less than the battery capacity *Q*, while when the car's charge is ${\bar{Y}}_{0}+\varepsilon$, the solution will not be optimal where *ε* is a small positive scalar. By Proposition 1, we can find in this case when the car is fully charged, the solution is also optimal, while ${\bar{Y}}_{0}+\varepsilon \leq Q$, the multiple optima exist.

**Proposition 2 .**

*If an optimal solution exists and there is a MCV that has been charged at least once and has extra power, that isy*_*n*+1_^*∗*^ > 0*, when it returns to the depot at the end of the service, then the solution that isy*_*n*+1_^*∗*^=0*, is also optimal*.

**Proof**

Let *y*_*n*+1_^*∗*^ > 0 be optimal. When recharging the battery with less energy will reduce the recharging time, which will not break any service time window, *y*_*n*+1_^*∗*^=0 must be an optimal solution.

**Corollary 2 .**

*If there is a MCV with extra power when it returns to the depot at the end of its route, that is* ${\bar{y}}_{n+1}>0$ *, and the solution is optimal, then there are infinite multiple optima for this problem*.

**Proof**

Using contradiction, let ${\bar{y}}_{n+1}>0$ be optimal and the solution ${\bar{y}}_{n+1}-\varepsilon$ not, where *ε* is a small positive scalar. Then, following Proposition 2, ${\bar{y}}_{n+1}-\varepsilon \geq y_{n+1}^{*}\geq 0$, so the multiple optima exist.

As the proposed formulation is a MILP, this model could be solvable in GAMS or other integer programming solvers.

## 4. Numerical Experiments

This section examines a large number of numerical experiments to demonstrate the model. The computing device used in this research is a personal computer with Intel(R) Core(TM) i7 6700U 3.40 GHz CPU and 16.00 GB·RAM, using the Microsoft Windows 7(64 bit) operation system. The general purpose optimization modeling package GAMS with high performance solver CPLEX is employed as the modeling tool in the numerical experiment [22]. Actually, GAMS is a high-level modeling system for optimization problems, which is tailored for complex, large scale modeling applications and designed for modeling linear, nonlinear, and mixed integer optimization problems. And CPLEX acts as a flexible, high-performance mathematical programming solver for linear programming, mixed integer programming, quadratic programming, and quadratically constrained programming problems. By taking advantage of commercial solver, we could solve the presented model.

This section is organized as follows: Section 4.1 describes the new designed instances based on the well-known VRPTW instances of Solomon [21]; our findings on the generated instances are presented in Section 4.2; and in Section 4.3, sensitivity analyses are conducted.

### 4.1. Generation of Benchmark Instances

To demonstrate the proposed model, a series of numerical experiments are conducted. As there are no benchmark instances for mobile charging vehicle routing procedure, here we generate 36 small instances based on the well-known VRPTW instances of Solomon [21]. Depending on geographical information of charging requests locations, the instances could be divided into 3 classes, where customer nodes are clustered (C), randomly distributed (R), and both clustered and randomly distributed (RC). In addition, we limit the possible locations in order to generate feasible and meaningful instances. For each customer, it should be visited by the mobile charging vehicles departing from the depot with at most two different charging stations. So, the charging stations are located within the area of a cycle with a radius at most triple running range of the mobile charging vehicles. Specifically, there is one charging station located at the depot. The location of the remaining stations is determined in a random manner. For the instances, there are 5, 10, and 15 charging requests included. Furthermore, there are two groups defined according to schedule horizon, specifically, Group 1 containing the instances with short scheduling horizons, while Group 2 containing the ones with long scheduling horizon. Then, for Group 1, more mobile charging vehicles will be requested to complete all charging services. Therefore, there are less mobile charging vehicles requested by customers for Group 2.

For the way that other parameters, such as the power demand, the battery capacity, and the recharging rate, are set, we use the same way as Cui et al. [13]. In this paper, the battery capacity of mobile charging vehicles is nine times the average value of the total power demand at the customer nodes. As the battery capacity of the mobile charging vehicles is larger, the charging rate *g* for them is set as triple of the average charging rate for BEVs at customer nodes, which could be calculated by dividing the service time by the total power demand. We show the parameter values of all the instances in supplementary materials.

### 4.2. Performance on All Instances

Table 2 presents an overview of the baseline results. The computing time limit for GAMS is set to be 7,200 seconds. From the table, we can see that all the instances can be solved completely within the time limit. So results shown in Table 2 are accurate. For the notations, #*V* denotes the number of mobile charging vehicle, TD denotes the distance traveled, and *N* denotes the number of charging stations used in the service procedure. By the column #*V*, we can find that more vehicles are requested and less charging station are used for Group 1.

### 4.3. Sensitivity Analyses

Since the larger battery capacity can help the mobile charging vehicle reduce its own charging frequency and the faster charging rate reduces its own charging time, the battery capacity and charging rate of the mobile charging vehicle may affect the efficiency of providing the charging service and then affect the routing strategy of mobile charging vehicles. Therefore, these two factors will be discussed in Sections 4.3.1 and 4.3.2, respectively. In the Section 4.3.3, the effect of service time on the results is further analyzed.

#### 4.3.1. The Sensitivity Analysis of Battery Capacity

For the battery capacity of the mobile charging vehicles, three different cases are explored with pregiven charging requests. They are (I) seven times the average value of the total power demand at the customer nodes (BC-Case I), (II) nine times the average value of the total power demand at the customer nodes (BC-Case II), and (III) eleven times the average value of the total power demand at the customer nodes (BC-Case III). The recharging rate *g* of the mobile charging vehicle is set as triple of the average charging rate for BEVs at customer nodes.

**Figure 3:**
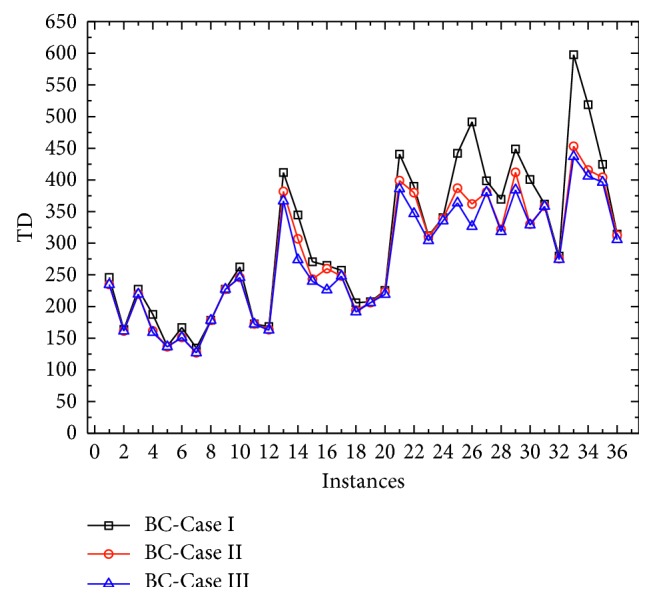
Numerical results for comparison with different battery capacity.

All sensitive analysis results for the battery capacity of mobile charging vehicles are shown in Table 3. From the table, we can find that when the battery capacity increases, the total travel distance will be decreasing, which can further be observed in Figure 3. As the object function is to minimize the total distance, with larger battery capacity, one mobile charging vehicle can service more customers without recharging demand. This is even more noticeable when the number of customer nodes is relatively large. Meanwhile, increasing the battery capacity can reduce the number of mobile charging vehicles and charging stations used. Nevertheless, there are still some abnormal points which have been bolded. The reason may be on the computation performance of commercial solver for some special problems.

#### 4.3.2. The Sensitivity Analysis of Recharging Rate

Next, we will discuss the sensitive analysis on the charging rate of the mobile charging vehicles.  Table 4 provides an overview of the corresponding results. Similarly, the following three different cases will be discussed, which are (I) the value of recharging rate *g* is equal to the average charge rate of the BEVs at customer node (CR-Case I), (II) the value *g* is 2 times of the average charge rate of the BEVs at customer node (CR-Case II), and (III) the value *g* is 3 times of the average charge rate of the BEVs at customer node (CR-Case III). The battery capacity of the mobile charging vehicles is nine times the average value of the total power demand at the customer nodes.

**Figure 4:**
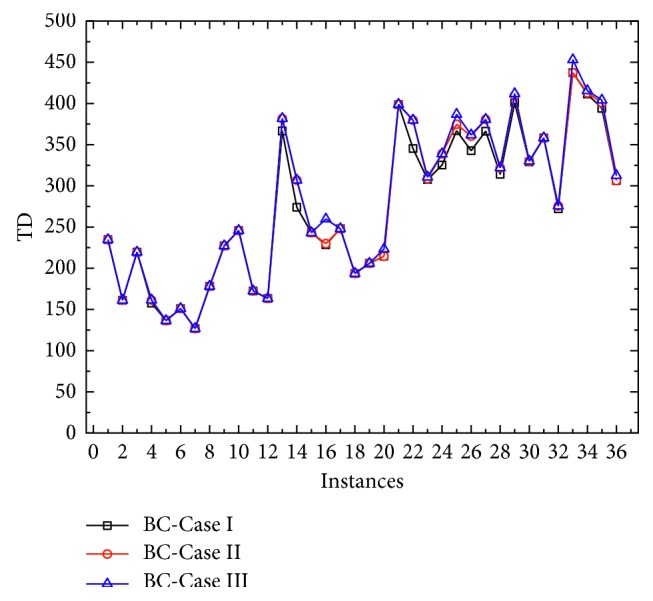
Numerical results for comparison with different charge rate.

In Table 4, it is obvious that as the charging rate increases, the number of mobile charging vehicles used and the total distance traveled decrease. We bold the results with changes and show the traveled distance results of all instances in three different cases in Figure 4. Interestingly, it seems that one could not check obvious variation of the number of used charging station as expected. The reason is that in order to minimize the total distance, the total amount of electricity supplied by all mobile charging vehicles may be greater than the total demand of the requesting BEVs, so the variation is relatively small with no obvious regularity.

#### 4.3.3. The Sensitivity Analysis of Service Time

As the results of Section 4.3.2 show, faster charging rates can improve service efficiency, reduce the total traveled distance, and even reduce the number of vehicles used. Similarly, if the charging service efficiency is improved and the service time at the customer node is reduced, the result of reducing the total travel distance can also be achieved, and even the number of vehicles can be reduced. Therefore, in this part, we assume a threefold increase in service efficiency in customer nodes, and then we test the results of the three different battery capacities shown in Section 4.3.1.

**Figure 5:**
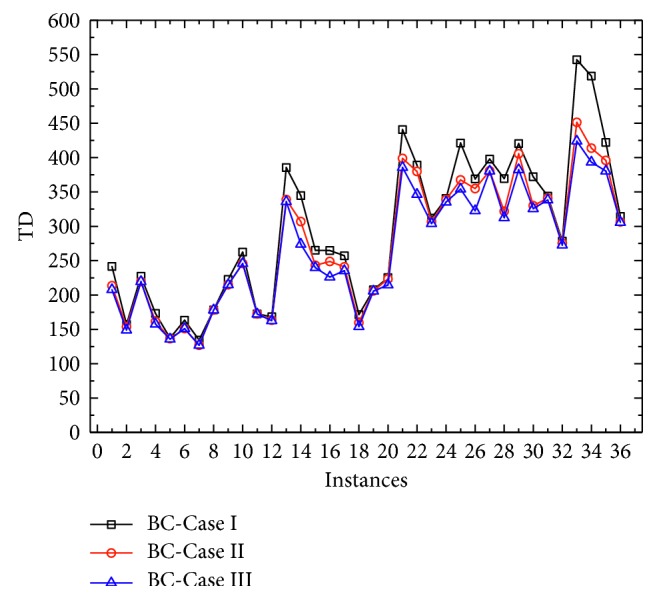
Numerical results for comparison with different battery capacity.

The corresponding results are shown in Table 5. We compare the #*V*, TD, and *N* columns for the three cases with the results shown in Table 3, and the improved results are marked in bold. For traveled distance, 18 solutions have been improved for BC-Case I, 16 solutions improved for BC-Case II, and 21 solutions improved for BC-Case III.  Figure 5 shows the traveled distance results of three cases.

## 5. Conclusions and Discussions

In this paper, a novel approach for routing mobile charging vehicles serving for BEVs with charging requests is proposed. An MILP formulation based on the VRPTW is presented. By taking advantage of the commercial solver, the problem could be solved. To demonstrate the model, 36 small instances are designed based on the benchmark instances for VRPTW proposed by Solomon [21]. All instances can be solved exactly, and sensitivity analyses are also conducted. It could be concluding that larger battery capacity, quicker charging rate, or higher service efficiency could decrease the number of mobile charging vehicles deployed and total traveled distance, respectively.

Though the model presented here is relatively simple, it shows the need for optimizing the mobile charging services from the operational research perspective, and a potential application of the classical VRPTW. Furthermore, several ways could be expanded for the model. To solve the problem with larger size, more efficient algorithms, such as the adaptive large neighborhood search (ALNS) algorithm and hybrid heuristic are expected. Moreover, future work will enhance the accuracy of the service model such as heterogeneity of both the mobile charging vehicles and the requests, grades of roads, and vehicle speeds of MCVs. Finally, dynamic service requests will change the service path of the vehicle at any time, which will be a more complicated research direction. Future studies could address the behavioral oriented charging services combined with vehicle routing.

## Figures and Tables

**Table 1:** Indices, sets, parameters and variables.

Indices	Definition
0, *N*+1	Index of depot instances and every route starts at 0 and ends at *N*+1
*i*, *j*	Index of nodes, *i*, *j* ∈ *V*_0,*N*+1_′
(*i*, *j*)	Index of physical link between two adjacent nodes, (*i*, *j*) ∈ *A*
*Sets*	
*F*	Set of recharging station
*F*′	Set of dummy vertices of *F*
*F*_0_′	Set of recharging visits including depot instance 0
*V*	Set of customers
*V*_0_	Set of customers including depot instance 0
*V*′	Set of nodes including customers and recharging stations, *V*′=*V* ∪ *F*′
*V*_0_′	Set of customers and recharging stations including depot instance 0, *V*_0_′=*V*′ ∪ {0}
*V*_*N*+1_′	Set of customers and recharging stations including depot instance *N*+1, *V*_*N*+1_′=*V*′ ∪ {*N*+1}
*V*_0,*N*+1_′	Set of customers and recharging visits including depot instance 0 and *N*+1, *V*_0,*N*+1_′=*V*′ ∪ {0} ∪ {*N*+1}
*Parameters*	
*d*_*ij*_	Distance of link (*i*, *j*) ∈ *A*
*t*_*ij*_	Travel time of link (*i*, *j*) ∈ *A*
*g*	Recharging rate
*q*_*i*_	The power demand for one electric car in node *i* ∈ *V*
*Q*	Battery capacity for mobile charging service car
*h*	Charge consumption rate in traveling
*u*_*i*_	Demand number of node *i* ∈ *V* and *u*_*i*_=0 if *i* does not belong to set *V*
*e*_*i*_	Earliest start of service at node *i* ∈ *V*
*l*_*i*_	Latest start of service at node *i* ∈ *V*, it is always a positive number of infinity
*s*_*i*_	Service time at node *i* ∈ *V*
*b*_*i*_	The node ∀*i* ∈ *V*_0,*N*+1_′ can provide the upper limit of electricity for recharging
*K*	The sufficiently large constant
*Variables*	
*τ*_*i*_	The time of arrive at node *i* ∈ *V*_0,*N*+1_′
*r*_*i*_	The recharging amount of electricity at node *i* ∈ *V*_0,*N*+1_′
*x*_*ij*_	=1 if arc (*i*, *j*) is traveled; =0 otherwise
*ρ*_*ij*_	=0 if arc (*i*, *j*) is utilized; otherwise, is unrestricted
*y*_*i*_	The remaining battery on arrival at node *i*

**Table 2:** Baseline results for the mobile charging vehicle routing problem.

Inst.	#*V*	TD	*N*
*C*101 − 5	3	234.72	30
*C*103 − 5	2	161.26	0
*C*206 − 5	2	219.54	2
*C*208 − 5	1	161.42	3
*R*104 − 5	2	136.45	0
*R*105 − 5	2	151.15	0
*R*202 − 5	1	126.83	1
*R*203 − 5	1	178.17	1
*RC*105 − 5	2	227.19	0
*RC*108 − 5	2	245.92	2
*RC*204 − 5	1	172.49	1
*RC*208 − 5	1	163.33	1
*C*101 − 10	4	381.69	2
*C*104 − 10	3	306.89	1
*C*202 − 10	2	243.19	2
*C*205 − 10	3	259.76	4
*R*102 − 10	3	248.00	2
*R*103 − 10	3	193.69	0
*R*201 − 10	3	206.05	2
*R*203 − 10	1	223.38	3
*RC*102 − 10	4	398.85	1
*RC*108 − 10	4	379.77	2
*RC*201 − 10	3	310.62	3
*RC*205 − 10	3	338.92	4
*C*103 − 15	4	386.88	4
*C*106 − 15	6	361.82	3
*C*202 − 15	3	380.64	5
*C*208 − 15	3	321.89	3
*R*102 − 15	6	411.91	2
*R*105 − 15	4	330.00	3
*R*202 − 15	3	358.28	3
*R*209 − 15	2	275.56	5
*RC*103 − 15	5	453.04	4
*RC*108 − 15	4	415.57	4
*RC*202 − 15	3	404.10	6
*RC*204 − 15	2	312.61	4

*Note.*#*V* denotes the vehicle number, TD the traveled distance, and *N* the number of charging stations used.

**Table 3:** Sensitive analysis on different battery capacity.

Inst.	BC − Case I			BC − Case II			BC − Case III		
	#*V*	TD	*N*	#*V*	TD	*N*	#*V*	TD	*N*
*C*101 − 5	3	245.89	2	3	234.72	0	3	234.72	0
*C*103 − 5	2	163.77	2	2	161.26	0	2	161.26	0
*C*206 − 5	2	227.33	4	2	219.54	2	2	219.54	2
*C*208 − 5	2	187.66	3	1	161.42	3	1	159.02	2
*R*104 − 5	2	137.32	1	2	136.45	0	2	136.45	0
*R*105 − 5	3	166.79	2	2	151.15	0	2	151.15	0
*R*202 − 5	1	134.41	2	1	126.83	1	1	126.83	1
*R*203 − 5	1	178.31	3	1	178.17	1	1	178.17	1
*RC*105 − 5	2	227.24	1	2	227.19	0	2	227.19	0
*RC*108 − 5	2	262.48	4	2	245.92	2	2	245.87	0
*RC*204 − 5	1	172.49	2	1	172.49	1	1	172.24	1
*RC*208 − 5	1	168.21	2	1	163.33	1	1	162.67	1
*C*101 − 10	5	411.53	3	4	381.69	2	3	366.65	2
*C*104 − 10	4	344.57	3	3	306.89	1	3	273.89	3
*C*202 − 10	2	270.74	6	2	243.19	2	2	239.84	0
*C*205 − 10	3	264.76	5	3	259.76	4	2	226.19	3
*R*102 − 10	4	257.16	4	3	248.00	2	3	247.93	0
*R*103 − 10	3	205.83	4	3	193.69	0	3	191.33	1
*R*201 − 10	3	207.53	2	3	206.05	2	3	206.05	2
*R*203 − 10	1	225.35	6	1	223.38	3	1	219.15	3
*RC*102 − 10	5	440.68	4	4	398.85	1	3	385.79	1
*RC*108 − 10	4	389.79	4	4	379.77	2	3	346.79	3
*RC*201 − 10	3	311.92	5	3	310.62	3	3	304.00	0
*RC*205 − 10	3	340.51	4	3	338.92	4	3	335.25	0
*C*103 − 15	5	441.91	7	4	386.88	4	4	363.58	2
*C*106 − 15	7	491.70	8	6	361.82	3	4	326.41	4
*C*202 − 15	4	398.42	7	3	380.64	5	3	380.44	5
*C*208 − 15	3	369.41	7	3	321.89	3	3	318.18	3
*R*102 − 15	6	448.81	6	6	411.91	2	5	384.17	3
*R*105 − 15	6	400.70	4	4	330.00	3	4	328.78	2
*R*202 − 15	3	361.74	5	3	358.28	3	3	358.15	2
*R*209 − 15	2	279.47	5	2	275.56	5	2	274.33	4
*RC*103 − 15	8	597.65	7	5	453.04	4	5	436.90	3
*RC*108 − 15	6	518.73	4	4	415.57	4	4	405.88	2
*RC*202 − 15	3	424.57	8	3	404.10	6	3	396.35	5
*RC*204 − 15	2	314.25	6	2	312.61	4	2	305.66	3
Average	3.25	305.27	4.22	2.81	280.04	2.31	2.61	272.13	1.78

*Note.*#*V* denotes the vehicle number, TD the traveled distance, and *N* the number of charging stations used.

**Table 4:** Sensitive analysis on different charge rate.

Inst.	CR − Case I(1)			CR − Case II(2)			CR − Case III(3)		
	#*V*	TD	*N*	#*V*	TD	*N*	#*V*	TD	*N*
*C*101 − 5	3	234.72	0	3	234.72	0	3	234.72	0
*C*103 − 5	2	161.26	0	2	161.26	0	2	161.26	0
*C*206 − 5	2	219.54	2	2	219.54	2	2	219.54	2
*C*208 − 5	1	157.72	1	1	161.42	3	1	161.42	3
*R*104 − 5	2	136.45	0	2	136.45	0	2	136.45	0
*R*105 − 5	2	151.15	0	2	151.15	0	2	151.15	0
*R*202 − 5	1	126.83	1	1	126.83	1	1	126.83	1
*R*203 − 5	1	178.17	1	1	178.17	1	1	178.17	1
*RC*105 − 5	2	227.19	0	2	227.19	0	2	227.19	0
*RC*108 − 5	2	245.92	2	2	245.92	2	2	245.92	2
*RC*204 − 5	1	172.49	1	1	172.49	1	1	172.49	1
*RC*208 − 5	1	163.33	1	1	163.33	1	1	163.33	1
*C*101 − 10	3	366.65	3	4	381.69	2	4	381.69	2
*C*104 − 10	2	273.89	2	3	306.89	1	3	306.89	1
*C*202 − 10	2	243.19	3	2	243.19	3	2	243.19	2
*C*205 − 10	2	228.50	3	2	229.87	4	3	259.76	4
*R*102 − 10	3	248.00	2	3	248.00	2	3	248.00	2
*R*103 − 10	3	193.69	0	3	193.69	0	3	193.69	0
*R*201 − 10	3	206.05	2	3	206.05	2	3	206.05	2
*R*203 − 10	1	214.61	3	1	214.61	3	1	223.38	3
*RC*102 − 10	4	398.62	2	4	398.85	1	4	398.85	1
*RC*108 − 10	3	345.35	2	4	379.77	2	4	379.77	2
*RC*201 − 10	2	307.72	4	2	308.59	4	3	310.62	3
*RC*205 − 10	2	325.27	4	3	338.92	4	3	338.92	4
*C*103 − 15	4	366.90	4	4	374.71	3	4	386.88	4
*C*106 − 15	4	342.68	5	5	360.34	4	6	361.82	3
*C*202 − 15	3	366.45	5	3	380.64	5	3	380.64	5
*C*208 − 15	3	313.99	4	3	321.89	3	3	321.89	3
*R*102 − 15	5	400.90	5	6	409.60	2	6	411.91	2
*R*105 − 15	4	329.05	4	4	330.00	3	4	330.00	3
*R*202 − 15	3	358.28	4	3	358.28	3	3	358.28	3
*R*209 − 15	2	272.14	4	2	275.56	5	2	275.56	5
*RC*103 − 15	5	437.29	4	5	437.29	4	5	453.04	4
*RC*108 − 15	4	411.35	4	4	412.80	4	4	415.57	4
*RC*202 − 15	2	394.11	7	3	400.95	6	3	404.10	6
*RC*204 − 15	2	306.37	5	2	306.37	5	2	312.61	4
Average	2.53	272.94	2.61	2.72	277.70	2.39	2.81	280.04	2.31

*Note.*#*V* denotes the vehicle number, TD the traveled distance, and *N* the number of charging stations used.

**Table 5:** Sensitive analysis on service efficiency.

Inst.	BC − Case I			BC − Case II			BC − Case III		
	#*V*	TD	*N*	#*V*	TD	*N*	#*V*	*TD*	*N*
*C*101 − 5	3	241.51	3	2	213.53	1	2	207.82	2
*C*103 − 5	2	157.29	3	2	153.75	1	1	149.04	1
*C*206 − 5	2	227.33	4	2	219.54	2	2	219.54	2
*C*208 − 5	1	173.08	3	1	161.42	3	1	157.72	2
*R*104 − 5	2	137.32	1	2	136.45	0	1	135.81	2
*R*105 − 5	2	163.36	4	2	151.15	1	2	151.15	0
*R*202 − 5	1	134.41	2	1	126.83	1	1	126.83	1
*R*203 − 5	1	178.31	3	1	178.17	2	1	178.17	1
*RC*105 − 5	2	222.68	3	2	215.00	0	2	215.00	0
*RC*108 − 5	2	262.48	4	2	245.92	2	2	245.87	0
*RC*204 − 5	1	172.49	2	1	172.49	1	1	172.24	1
*RC*208 − 5	1	168.21	2	1	163.33	1	1	162.67	1
*C*101 − 10	4	385.56	5	3	338.95	4	3	335.95	0
*C*104 − 10	4	344.57	3	3	306.89	2	2	273.89	3
*C*202 − 10	2	265.11	6	2	243.19	2	2	239.84	1
*C*205 − 10	3	264.76	4	2	248.82	4	2	226.19	1
*R*102 − 10	4	257.16	3	3	240.91	2	3	235.45	1
*R*103 − 10	2	171.54	4	2	159.63	3	2	154.04	0
*R*201 − 10	3	207.53	3	3	206.05	1	3	205.60	2
*R*203 − 10	1	225.35	5	1	223.38	3	1	214.61	2
*RC*102	5	440.68	5	4	398.85	1	3	385.79	1
*RC*108	4	389.10	5	4	379.77	2	3	346.40	3
*RC*201	3	311.92	5	3	306.55	3	3	304.00	1
*RC*205	3	340.51	4	3	338.92	4	3	335.25	1
*C*103 − 15	5	421.20	6	4	367.55	2	4	354.24	2
*C*103 − 15	6	369.02	5	6	354.85	3	4	322.59	3
*C*202 − 15	4	397.82	5	3	380.64	5	3	380.44	5
*C*208 − 15	3	369.41	7	3	321.89	3	3	312.48	3
*R*102 − 15	6	420.32	6	5	405.56	4	5	382.42	3
*R*105 − 15	5	372.06	7	4	330.00	3	4	325.68	3
*R*202 − 15	3	344.02	6	3	341.02	3	2	338.37	5
*R*209 − 15	2	278.37	5	2	275.56	5	2	272.72	4
*RC*103 − 15	6	542.42	9	5	451.30	5	5	423.96	4
*RC*108 − 15	6	518.73	4	4	413.78	3	3	393.39	5
*RC*202 − 15	3	421.99	8	2	395.90	8	2	380.40	6
*RC*204 − 15	2	314.25	6	2	306.37	5	2	305.66	3
_Average	3.03	294.77	4.44	2.64	274.28	2.64	2.39	265.87	2.08

*Note.*#*V* denotes the vehicle number, TD the traveled distance, and *N* the number of charging stations used.

## Data Availability

The data used to support the findings of this study are available from the corresponding author upon request.

## Supplementary Materials

Supplementary Materials All benchmark instances presented in this paper are shown in Folder Section 4.2. The name of each text file uses the corresponding example name, such as RC108-5. Specifically, all related data, such as location of charging stations, time windows, service time, and so on, are contained. Folder Section 4.3 contains three folders, named as Section 4.3.1, Section 4.3.2, and Section 4.3.3, which contain the data requirements of the corresponding part of the paper.
